# Application of the Singular Spectrum Analysis Technique to Study the Recent Hiatus on the Global Surface Temperature Record

**DOI:** 10.1371/journal.pone.0107222

**Published:** 2014-09-10

**Authors:** Diego Macias, Adolf Stips, Elisa Garcia-Gorriz

**Affiliations:** European Commission, Joint Research Centre, Institute for Environment and Sustainability, Water Research Unit, Ispra, Italy; University of Oxford, United Kingdom

## Abstract

Global surface temperature has been increasing since the beginning of the 20^th^ century but with a highly variable warming rate, and the alternation of rapid warming periods with ‘hiatus’ decades is a constant throughout the series. The superimposition of a secular warming trend with natural multidecadal variability is the most accepted explanation for such a pattern. Since the start of the 21^st^ century, the surface global mean temperature has not risen at the same rate as the top-of-atmosphere radiative energy input or greenhouse gas emissions, provoking scientific and social interest in determining the causes of this apparent discrepancy. Multidecadal natural variability is the most commonly proposed cause for the present hiatus period. Here, we analyze the HadCRUT4 surface temperature database with spectral techniques to separate a multidecadal oscillation (MDV) from a secular trend (ST). Both signals combined account for nearly 88% of the total variability of the temperature series showing the main acceleration/deceleration periods already described elsewhere. Three stalling periods with very little warming could be found within the series, from 1878 to 1907, from 1945 to 1969 and from 2001 to the end of the series, all of them coincided with a cooling phase of the MDV. Henceforth, MDV seems to be the main cause of the different hiatus periods shown by the global surface temperature records. However, and contrary to the two previous events, during the current hiatus period, the ST shows a strong fluctuation on the warming rate, with a large acceleration (0.0085°C year^−1^ to 0.017°C year^−1^) during 1992–2001 and a sharp deceleration (0.017°C year^−1^ to 0.003°C year^−1^) from 2002 onwards. This is the first time in the observational record that the ST shows such variability, so determining the causes and consequences of this change of behavior needs to be addressed by the scientific community.

## Introduction

The record of global mean temperature anomalies (GMTA), available at monthly resolution since 1850, is a noisy time series that is complicated to analyze due to the large number of forcing factors. Among the different contributions, two basic modes of variability stand out from the rest [1], [2]: a secular trend (ST) with no obvious oscillation cycles [3]–[6], and a multidecadal variability (MDV) resembling natural oscillations such as the Pacific Multidecadal Oscillation (PDO) [7] or the Atlantic Multidecadal Oscillation (AMO) [8], [9]. Furthermore, there are numerous internal and external forcings that contribute to climate variability, such as solar cycles [10], stratospheric sulfur aerosols [11] or stratospheric water vapor [12], at a much higher frequency.

The combination of ST + MDV drives the main dynamics of the GMTA series [3], drawing a multidecadal variation over a background trend of increasing temperature. The alternation of periods with accelerated warming rates with others having lower rates, were described almost 30 years ago [1], whereas the actual temperature deceleration period (so called *hiatus*) had already been predicted 20 years ago [2]. Most of the recent research tends to agree with natural variability being the most probable cause of the last 15-year hiatus in surface temperature [4], [5] as the MDV is currently on a negative phase [7]. An increased storage of heat in the deep ocean is one of the most recurrent explanations derived from modeling exercises [8], [13] and from measurement records [14], [15]; negative tropical sea surface temperature anomalies have also been recently invoked as a major forcing for the recent warming slow-down [16]. However, a global consensus on the causes of the present-day hiatus is still far from being reached.

The identification and attribution of the different modes of variability is a recurrent problem in temperature time-series analysis, and different methods have been employed to discriminate the different signals contributing to the total variability of the series [9], [17]. An adequate analysis method should be non-parametric and objective and thus not depend on the choice of the included processes [9]. Singular spectrum analysis (SSA) fulfills these requisites and hence has been successfully employed for analyzing geophysical time series, such as air temperature [2] and sea surface temperature [18].

## Results and Discussion

**Figure 1:**
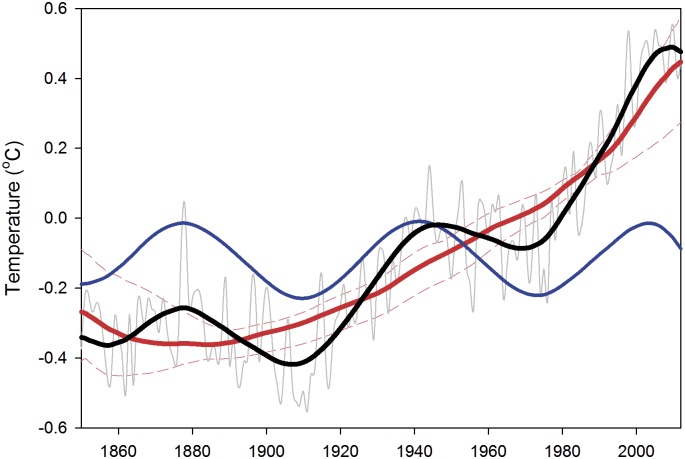
SSA reconstructed signals from HadCRUT4 global surface temperature anomalies. The annual surface temperature (gray line), multidecadal variability (MDV, blue line), secular trend (ST, red line) and reconstructed signal (MDV+ST, black line) are indicated. ST represents 78.8% of the total energy of the series; MDV accounts for 8.8% of the energy and the reconstructed signal for 88%. The dashed thin red lines indicate the range of variability of the ST obtained by applying SSA to the temperature time series obtained for each individual month.

**Figure 2:**
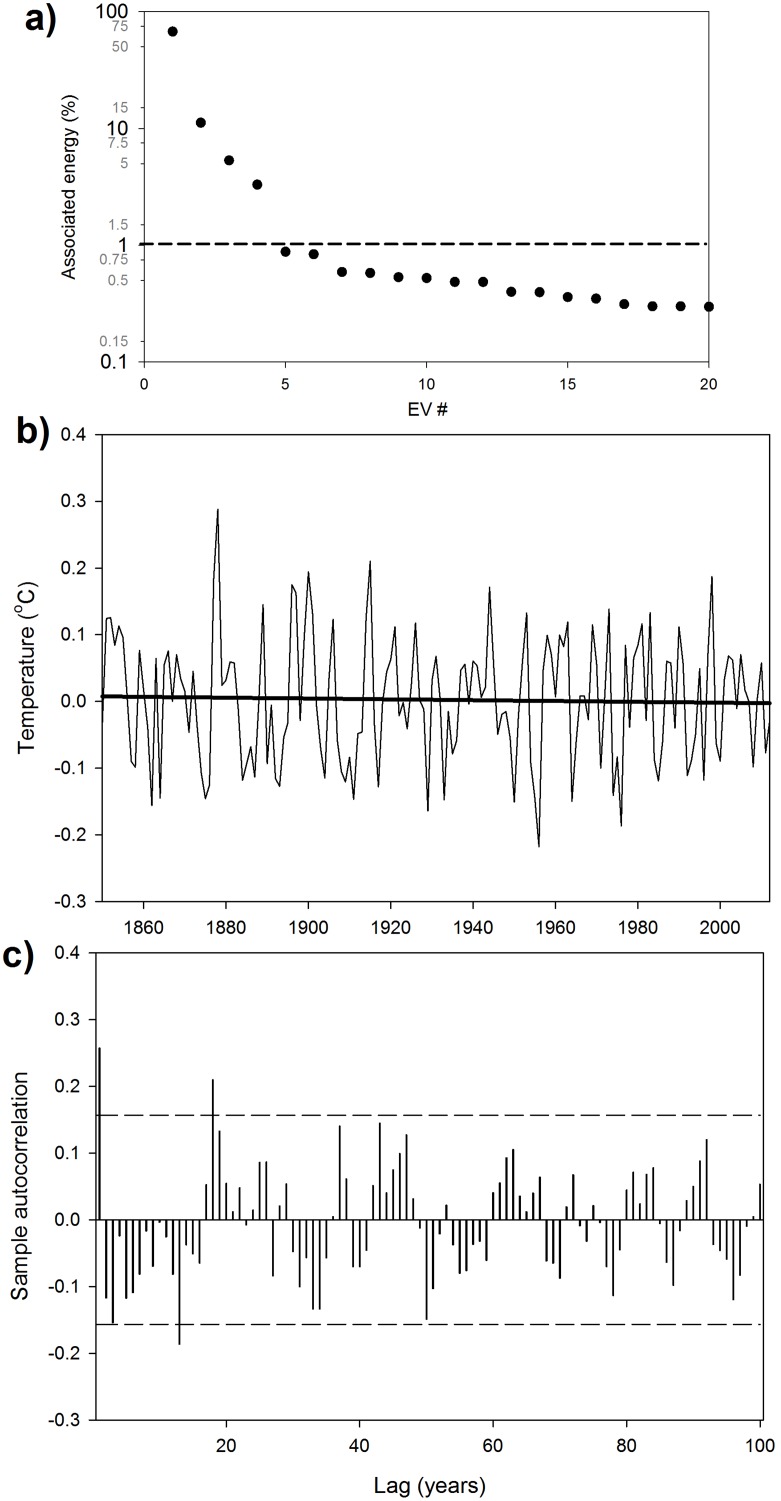
Singular spectrum analysis of the GMTA. a) Energy diagram of the first 20 eigenvectors (EV) obtained from the SSA. The horizontal dashed line indicates the 1% energy limit. b) Residuals obtained by removing the signals associated with the first four EV from the original GMTA record. The black bold line represents the linear fit (not significant, p>0.05). c) Autocorrelation analysis of the residuals. The horizontal dashed lines indicate significant bounds.

By applying SSA to the latest surface temperature database (HadCRUT4) at annual resolution, it is found that nearly 88% of the total variability of GMTA is due to the combination of ST (78.8%) and MDV (8.6%) [4], [6] (Fig. 1). ST is obtained from the combination of eigenvectors (EVs) #1 + #2, while MDV is the result of EVs #3 + #4 (Fig. 2a).

The remaining 12% of the series contain very little information, with no significant trend and almost no autocorrelation (Fig. 2), so it could be considered as white noise. Higher-frequency oscillations (such as those associated with solar activity) [10] are contained in the residuals; e.g., EVs #7 and #8 define a 13-year period signal, while EVs #9 and #10 define an 18-year period signal (also identified through the slightly significant autocorrelation values at time lags 13 and 18 years in Fig. 2c). However, as their contribution to the total signal is rather weak (below 1%, Fig. 2a) and their oscillation period is rather short, it is acceptable not to consider them in subsequent long-term analyses [17]. The presence of these higher-frequency cycles in the residuals is, however, evidence of the capability of SSA to separate the signals existing in the original time-series.

**Figure 3:**
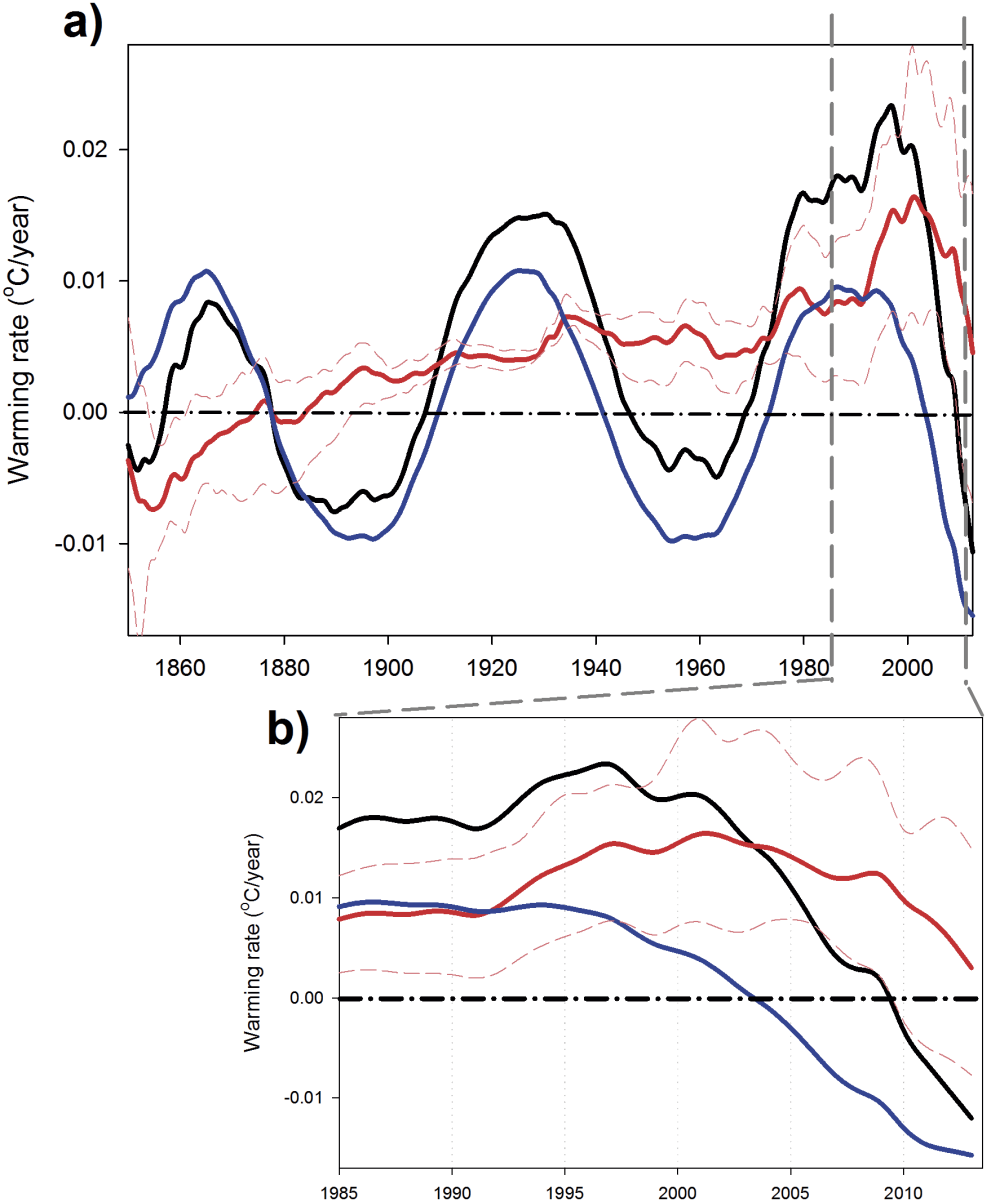
Global warming rate analysis. a) Warming rates (°C year^−1^) obtained from the different signals identified in the SSA: ST (red line), MDV (blue line) and reconstructed signal (black line). The dashed thin red lines are the confidence intervals for the warming rate associated with the ST obtained from each individual month’s time series. b) Zoom on the last 25 years of the time series.

The reconstructed surface temperature series obtained by adding ST+MDV (black line in Fig. 1) presents three hiatus periods with no warming or even with small cooling trends, 1878–1907; 1945–1969 and 2001–2013, roughly one every 62 years [19]. This is more noticeable if the annual gradient of each signal is plotted against time (Fig. 3). Obviously in this figure, the stalling periods of the reconstructed series are coincident with cooling trends in the MDV. Results of our analysis are, henceforth, clearly suggesting that natural climate variability is a key player in global temperature hiatus periods, as previously suggested [4], [5]. On the other hand, when MDV is on a positive phase (e.g., from 1970 to 1995) the total warming rate greatly accelerates (Fig. 3) as also suggested in previous analysis [19], [20].

On the background, the ST has shown positive warming rates since the beginning of the 20^th^ century (Fig. 3a, red line) with an increasing trend up to the final years of the 1990’s. It is also clear that the ST continued to increase during the two first hiatus events (Fig. 1), showing small variations of its warming trend (Fig. 3a). However, the current hiatus is somehow different (details in Fig. 3b). After the maximum warming rate associated with MDV was reached by approximately 1990, ST showed a distinct peak from 1992–2001, with an unprecedented increase of its warming rate from 0.0085°C year^−1^ to 0.017°C year^−1^, almost doubling in one decade. After this warming rate peak, the ST shows a pronounced decline, 0.017°C year^−1^ in 2001 to 0.003°C year^−1^, in 2013. This type of quick fluctuations in the ST warming rate has no precedent in the observational record (Fig. 3a). Thus, our analysis appears to indicate that the recent hiatus in global surface air temperature is mostly attributable to a negative phase of the MDV (as the two previous ones) plus a reduction of the warming rate associated with the ST (but still showing positive values).

The fluctuations observed in the warming rate associated with the ST during the last 20 years (1992–2013) could not, in principle, be due to a statistical artifact of the analysis method, the so-called ‘end-effect’, because of the underlying mathematics in such method. However, and in order to assess the existence of such a problem, we applied the same SSA analysis described below (see Material and Methods) to a fraction of the total GMTA from 1850 to 1960 when the previous hiatus was present (see Fig. 1). In this analysis (not shown) the ST did not display any variations at the end of the series so the recent fluctuation could not be attributed to a methodological problem with the used statistics but rather seems to correspond to a distinct dynamics of the observed temperature series. However, the relatively short time-period in which such fluctuation happened (∼20 years) prevents more solid conclusions to be derived.

**Figure 4:**
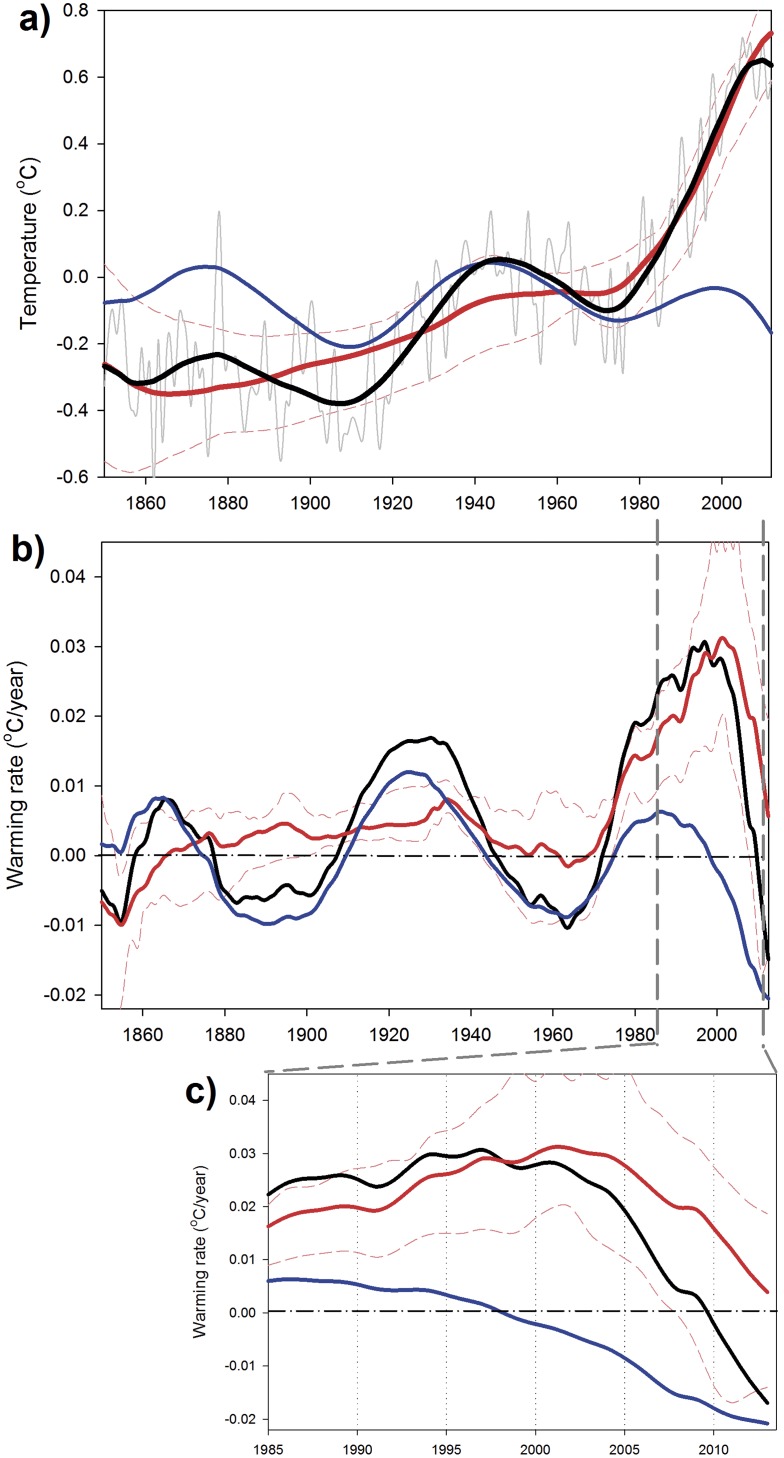
SSA reconstructed signals for Northern Hemisphere surface temperature. a) HadCRUT4 annual surface temperature (gray line), multidecadal variability (MDV, blue line), secular trend (ST, red line) and reconstructed signal (MDV+ST, black line). The dashed thin red lines indicate the range of variability of the ST obtained by applying SSA to the temperature time series obtained for each individual month. b) Warming rates (°C year^−1^) obtained from the different signals identified in the SSA for the Northern Hemisphere. The dashed thin red lines are the confidence intervals for the warming rate associated with the ST obtained from each individual month’s time series. c) Zoom on the last 25 years of the warming rate time series.

**Figure 5:**
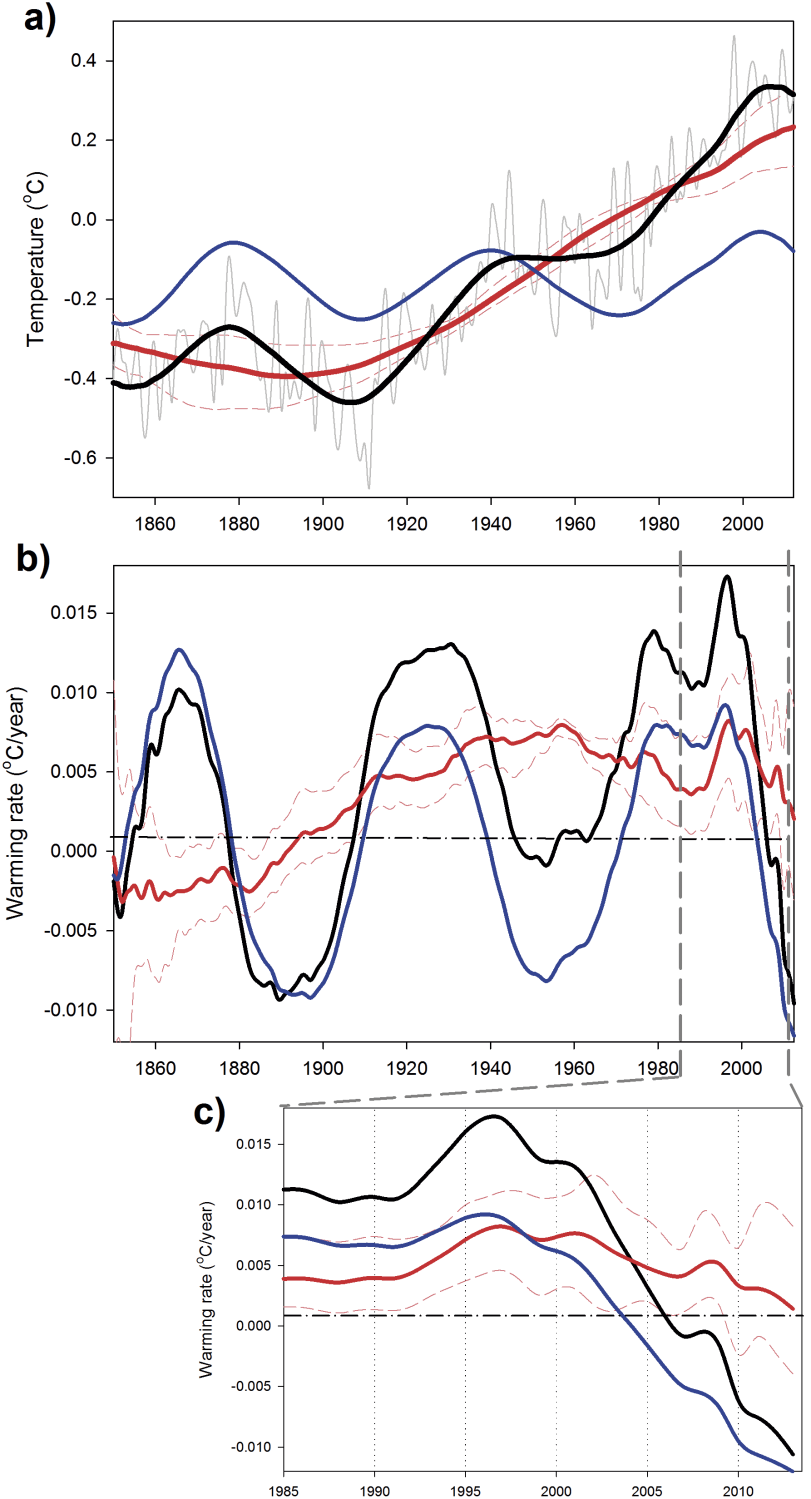
SSA reconstructed signals for Southern Hemisphere surface temperature. a) HadCRUT4 annual surface temperature (gray line), multidecadal variability (MDV, blue line), secular trend (ST, red line) and reconstructed signal (MDV+ST, black line). The dashed thin red lines indicate the range of variability of the ST obtained by applying SSA to the temperature time series obtained for each individual month. b) Warming rates (°C year^−1^) obtained from the different signals identified in the SSA for the Southern Hemisphere. The dashed thin red lines are the confidence intervals for the warming rate associated with the ST obtained from each individual month’s time series. c) Zoom on the last 25 years of the warming rate time series.

Analyzing surface air temperature data from the different hemispheres with the same technique gives results that are quite similar to those obtained for the global data (Figs. 4 and 5). Three cooling periods associated with cooling phases of the MDV are observable in the records at the same time as those detected in the global dataset (Fig. 3). In these separate datasets, periods of acceleration in the warming rate associated with the ST could be observed before the present day (e.g., 1855–1860 in the Northern Hemisphere and 1890–1910 in the Southern Hemisphere) as well as some deceleration periods (e.g., 1940–1967 in the Northern Hemisphere and 1960–1980 in the Southern Hemisphere) (Figs. 4 and 5). However, the magnitudes of the fluctuations are much larger in the period 1990–2013, also happening in a much shorter time-scale.

Therefore, the very recent strong changes observed in the warming rate associated with the ST appear to be a global phenomenon that had not occurred before (at least not during the last 160 years). It could not be attributable to MDV or any other form of climatic variability (such as solar cycles), as the different contributions are effectively separated by the SSA analysis (Fig. 2). This unprecedented modification of the ST behavior should be more deeply studied by the scientific community in order to address whether a change in the global climate sensitivity [21] has recently occurred.

The unique fluctuation in ST warming rate during the recent decades could have different origins, such as the proposed recent shift of the tropical Pacific Ocean to a quasi-permanent La Nina condition [16], could be related to the enhanced melting of Arctic ice in recent decades [22] or to the increasing stratospheric aerosol content [11]. A spatially-explicit analysis of the characteristics of the ST and MDV could, potentially, serve to better identify the likely mechanisms involved. Unfortunately, a 2D analysis could not be performed on the gridded HadCRUT4 dataset due to the sensibility of SSA to the presence of gaps in the analyzed data. Such gaps become more common when the studied area reduces preventing, thus, the analysis of the spatial distribution of the different signals.

Such an analysis could have also helped in clarifying the relation of the MDV to some of the previously described natural modes of surface temperature variability (e.g., PDO and AMO). However, a simple phase analysis (not shown) seems to indicate that the MDV is closer to AMO than to the PDO.

While it is beyond the scope of the present paper to elucidate the causes of the observed variability in the ST of surface temperatures, its unique presence in the entire observational time-series deserves some more detailed analysis in the coming future. Further studies on the roles and interplays of the atmosphere, the cryosphere and the hydrosphere are necessary to understand the causes of the observed changes. Such studies are crucial to being able to develop models that allow a realistic representation of the present day conditions [13], [20] and hence to create plausible scenarios of future climate evolution [5].

## Materials and Methods

### Surface temperature data

Mean surface air temperature anomalies were obtained from the HadCRUT4 dataset [23] (available at http://www.cru.uea.ac.uk/cru/data/temperature/, data downloaded on 01/2014). Different datasets spanning the period 1850–2013 were obtained: global mean temperature, temperatures of the Southern and Northern Hemispheres and the gridded dataset at 5°×5° resolution.

### Singular Spectrum Analysis (SSA)

Singular spectrum analysis (SSA) is a methodology specifically designed to extract information from noisy time series [24] and is analogous to applying an extended empirical orthogonal function (EEOF) analysis to successive lags of a univariate time series [25]. The particular SSA applied here was performed with the *Rssa* package of the statistical software **R**, freely available from the Comprehensive R Archive Network (CRAN, http://cran.r-project.org/). Using such a freely available code supports the reproducibility of the analysis performed in the present manuscript [26]. SSA allows decomposition of the time series into a sequence of elementary patterns of behavior that are classified as either trends (ST in this work) or oscillatory patterns. SSA was applied to the annual temperature anomalies in the different studied regions and to the different time series obtained for individual months. The latter analysis allows for the determination of whether the observed ST presents some type of seasonality and also allows for the computation of confidence intervals around the mean, as shown in the corresponding figures. In total, 81 eigenvectors were determined from each time series (of 161 years) using the Broomhead/King method to calculate covariance matrices [27].

## References

[1] Folland CK, Parker DE, Kates FE (1984) Worldwide marine temperature fluctuations 1856–1981. Nature 310, 670–673.

[2] Schlesinger ME, Ramankutty N (1994) An oscillation in the global climate system of period 65–70 years. Nature 367, 723–726.

[3] Stocker T, Dahe Q, Plattner G-K (coordinating lead authors) (2013) Intergovernmental Panel on Climate Change. Approved Summary for Policymakers of full draft report of Climate Change 2013: Physical Science Basis. Available: http://www.ipcc.ch/report/ar5/wg1/#.U1CweRCvHMM.

[4] Wu Z, Huang NE, Long SR, Peng CK (2007) On the trend, detrending, and variability of nonlinear and nonstationary time series. Proc. Natl. Acad. Sci. USA 104, 14889–14894. 10.1073/pnas.0701020104 PMC1986583 17846430

[5] Hunt BG (2011) The role of natural climatic variation in perturbing the observed global mean temperature trend. Clim. Dyn. 36, 509–521.

[6] Guemas V, Doblas-Reyes FJ, Andreu-Burillo I, Asif M (2013) Retrospective prediction of the global warming slowdown in the past decade. Nature Climate Change 3, 649–653.

[7] Meehl GA, Arblaster JM, Fasullo JT, Hu A., Trenberth KE (2011) Model-based evidence of deep-ocean heat uptake during surface-temperature hiatus periods. Nature Climate Change 1, 360–364.

[8] Keenlyside NS, Latif M, Jungclaus J, Kornblueh L, Roeckner E (2008) Advancing decadal-scales climate prediction in the North Atlantic sector. Nature 453, doi:10.1038/nature06921. 10.1038/nature06921 18451859

[9] Wu Z, Huang NE, Wallace JM, Smoliak BV, Chen X (2011) On the time-varying trend in global-mean surface temperature. Clim. Dyn. 37, 759–773.

[10] Kaufmann RK, Kauppi H, Mann ML, Stock JH (2011) Reconciling anthropogenic climate change with observed temperature 1998–2008. Proc. Natl. Acad. Sci. USA 108, doi:www.pnas.org/cgi/doi/10.1073/pnas.1102467108. 10.1073/pnas.1102467108 PMC3142005 21730180

[11] Solomon S, Daniel JS, Neely RR, Vernier JP, Dutton EG, et al. (2011) The Persistently Variable “Background” Stratospheric Aerosol Layer and Global Climate Change. Science 333, 866–870. 10.1126/science.1206027 21778361

[12] Solomon S, Rosenlof KH, Portmann RW, Daniel JS, Davis SM, et al. (2010) Contributions of stratospheric water vapor to decadal changes in the rate of global warming. Science 327, 1219, doi:10.1126/science.1182488. 10.1126/science.1182488 20110466

[13] Watanabe M, Kamae Y, Yoshimori M, Oka A, Sato M, et al. (2013) Strengthening of ocean uptake efficiency associated with the recent climate hiatus. Geophys. Res. Lett. 40, 3175–3179.

[14] Levitus S, Antonov JI, Boyer TP, Baranova OK, Garcia HE, et al. (2012) World ocean heat content and thermosteric sea level change (0–2000 m), 1955–2010. Geophys. Res. Let. 39, L10603, doi:10.1029/2012GL051106.

[15] Lyman JM, Good SA, Gouretski VV, Ishii M, Johnson JC, et al. (2010) Robust warming of the global upper ocean. Nature 465, doi:10.1038/nature09043. 10.1038/nature09043 20485432

[16] Kosaka Y, Xie SP (2013) Recent global-warming hiatus tied to equatorial Pacific surface cooling. Nature 12534. 10.1038/nature12534 23995690

[17] Santer BD, Mears C, Doutriaux C, Caldwell P, Gleckler PJ, et al. (2011) Separating signal and noise in atmospheric temperature changes: The importance of timescale. J. Geophys. Res. 116, D22105, doi:10.1029/2011JD016263.

[18] Macias D, Garcia-Gorriz E, Stips A (2013) Understanding the Causes of Recent Warming of Mediterranean Waters. How Much Could Be Attributed to Climate Change? Plos One 8, e81591. 10.1371/journal.pone.0081591 PMC3842300 24312322

[19] Meehl GA, Hu A, Arblaster JM, Fasullo J, Trenberth KE (2013) Externally forced and internally generated decadal climate variability associated with the Interdecadal Pacific Oscillation. J. Climate 26, 7298–7310.

[20] Fyfe JC, Gillet NP, Zwiers FW (2013) Overestimated global warming over the past 20 years. Nature Climate Change 3, 767–769.

[21] Hansen J, Kharecha P, Sato M, Masson-Delmonte V, Ackerman F, et al. (2013) Assessing “Dangerous Climate Change”: Required Reduction of Carbon Emissions to Protect Young People, Future Generations and Nature. Plos One 8, e81648. 10.1371/journal.pone.0081648 PMC3849278 24312568

[22] Stroeve JC, Serreze MC, Holland MM, Kay JE, Malanik J, et al. (2012) The Artićs rapidly shrinking sea ice cover: a research synthesis. Clim. Change 110, 1005–1027, doi:10.1007/s10584-011-0101-1.

[23] Morice CP, Kennedy JJ, Rayner NA, Jones P (2012) Quantifying uncertainties in global and regional temperature change using an ensemble of observational estimates: the HadCRUT4 dataset. J. Geophys. Res. 117, D08101.

[24] Ghil M, Allen MR, Dettinger MD, Ide K, Kondrashov D, et al. (2002) Advanced spectral methods for climatic time series. Rev. Geophys. 40, 1–41.

[25] Vautard R, Yiou P, Ghil M (1992) Singular Spectrum Analysis: A toolkit for short noisy chaotic signals. Phys. D. 58, 95–126.

[26] Añel JA (2011) The Importance of Reviewing the Code. Communications of the ACM, 54(5), 40–41.

[27] Broomhead DS, King GP (1986) Extracting qualitative dynamics from experimental data. Phys. D. 20(2–3), 217–236.

